# Response Surface Optimisation for the Production of Antioxidant Hydrolysates from Stone Fish Protein Using Bromelain

**DOI:** 10.1155/2017/4765463

**Published:** 2017-10-04

**Authors:** Shehu Muhammad Auwal, Mohammad Zarei, Azizah Abdul-Hamid, Nazamid Saari

**Affiliations:** ^1^Department of Food Science, Faculty of Food Science and Technology, Universiti Putra Malaysia, 43400 Serdang, Selangor, Malaysia; ^2^Department of Biochemistry, Faculty of Basic Medical Sciences, Bayero University, Kano, Nigeria; ^3^Department of Food Science and Technology, College of Agriculture and Natural Resources, Sanandaj Branch, Islamic Azad University, Sanandaj, Iran

## Abstract

Protein hydrolysates produced from different food sources exhibit therapeutic potential and can be used in the management of chronic diseases. This study was targeted to optimise the conditions for the hydrolysis of stone fish protein to produce antioxidant hydrolysates using central composite design (CCD) by response surface methodology (RSM). The stone fish protein was hydrolysed under the optimum predicted conditions defined by pH (6.5), temperature (54°C), E/S ratio (1.5%), and hydrolysis time (360 min). The hydrolysates were then evaluated for 2,2-diphenyl-1-picrylhydrazyl radical (DPPH^•^) scavenging activity and ferrous ion- (Fe^2+^-) chelating activity. Results validation showed no significant difference between the experimental values of DPPH^•^ scavenging activity (48.94%) and Fe^2+^-chelating activity (25.12%) obtained at 54.62% degree of hydrolysis (DH) compared to their corresponding predicted values of 49.79% and 24.08% at 53.08% DH, respectively. The hydrolysates demonstrated non-Newtonian behavior (*n* < 1) with stronger shear-thinning effect and higher viscosities at increasing concentration. Thus, RSM can be considered as a promising strategy to optimise the production of stone fish protein hydrolysates containing antioxidant peptides. It is hoped that this finding will enhance the potential of stone fish protein hydrolysates (SHs) as therapeutic bioactive ingredient in functional foods development.

## 1. Introduction

The global prevalence of chronic diseases related to free radical generation has necessitated the search for alternative approaches to the use of synthetic agents that are often costly and associated with one or more life-threatening side effects. Alternatively, protein hydrolysates containing potent antioxidant peptides have been produced from locally available and easily accessible food sources. In addition to the management of chronic diseases, these naturally occurring hydrolysates are safe and can be incorporated as additives to halt lipid peroxidation so as to improve the quality and consumer acceptability of many food products. According to Benjakul et al. [1]; McCarthy et al. [2], and Bhaskar et al. [3], protein hydrolysates possess high nutritional and therapeutic potentials with diverse dietary applications. In this study, the enzymatic production of antioxidant hydrolysates from stone fish protein has been successfully optimised making it a chief source of bioactive peptides for various applications.

Stone fish* (Actinopyga lecanora)* as one of the noble sources of antioxidant hydrolysates is a marine invertebrate from the phylum echinoderm and class Holothuroidea. It is identified among the edible species of sea cucumber commonly found in Malaysia and other south Asian countries mostly collected as bycatch of fishery industry [4, 5].

Bordbar et al. [6] and Lakshmi and Ghosal [7] have recently reported the presence of antioxidants and antiamoebic compounds in stone fish extracts. Stone fish hydrolysates with antihypertensive, antibacterial [5, 8], and antioxidant/free radical-scavenging activities [6] have been enzymatically generated under different reaction conditions. The antioxidant activity of stone fish hydrolysates was found to be dependent on the enzyme specificity, DH, and peptide sequences generated [8]. Thus, the selection of appropriate condition for a particular enzyme is crucial for obtaining maximum antioxidant activity of the hydrolysates.

The hydrolysis conditions of many food protein derived antioxidant hydrolysates have been optimised using RSM. RSM refers to a collection of statistical techniques used for model building to determine a combination of factor levels that produce an optimum response under the influence of one or more independent factor/s such as temperature, pH, time, and E/S ratio [9, 10]. It uses polynomial equation to explore the type of relationship between the factors and their main and combined effects on the desired response [11–13].

Examples include bovine plasma protein hydrolysates [14], jellyfish (*Rhopilema esculentum)* umbrella collagen hydrolysates [15], sheep visceral protein hydrolysates [16], Whey protein hydrolysates [11, 17], cuttlefish* (Sepia officinalis)* protein hydrolysates [18], and hydrolysates of pumpkin seed protein isolates [19].

However, the optimum conditions for the hydrolysis of stone fish protein in the production of antioxidant hydrolysates have not been established. Therefore, the present study was aimed at hydrolysing stone fish protein with bromelain according to CCD by RSM and study the effect of the process conditions including pH, temperature, E/S ratio and time on DH, DPPH radical-scavenging activity, and Fe^2+^-chelating activity of the resulting hydrolysates. The optimum levels of these conditions for the generation of hydrolysates with maximum antioxidant activities were also determined. Furthermore, the effect of shear rate and different concentrations of SHs on its viscosity have been studied.

## 2. Materials and Methods

Fresh sample of stone fish was obtained from Kedah and Langkawi Breeding Centers (Malaysia), bromelain from pineapple stem tissue, 2.4 to 3 U/mg, was obtained from Acros Organics (Geel, Belgium), 2,2-diphenyl-1-picrylhydrazyl (DPPH) was purchased from Sigma-Aldrich (St. Louis, MO, USA), iron (II) chloride tetrahydrate was obtained from Merck KGaA (Darmstadt, Germany), and all other chemicals used were of analytical grade and obtained from Acros Organics (Geel, Belgium), Fisher Scientific (Loughborough, Leics, UK), and J.T Baker (Thailand).

### 2.1. Preparation of Antioxidant Stone Fish Protein Hydrolysates (SHs) with Bromelain

The freeze-dried stonefish tissue was hydrolysed using bromelain as previously described by Auwal et al. [20]. Briefly, ten grams of the stone fish powder was transferred into a 12–14 kDa molecular mass cut-off dialysis tube and dialysed for 4 hours using deionised water as the suspending medium at room temperature and for 20 hours by replacing the deionised water with either of the reaction buffers (0.05 M acetate buffer pH 4, 0.05 M acetate buffer pH 5.5, or 0.05 M phosphate buffer pH 7) at 4°C (Table 1). After dialysis, the samples were mixed with 50 mL of the same reaction buffer and then preheated to the required temperature before enzyme addition (Table 1). The hydrolysis reaction was carried out at 150 rpm in water bath shaker. The enzyme was inactivated to terminate the reaction by heating the mixture in boiling water at 100°C for 10 minutes. After centrifugation at 4°C and 10,000 ×g for 20 min, the hydrolysates consisting of the antioxidant peptides were collected as the supernatant, then freeze dried, and stored at −40°C prior to analysis.

### 2.2. Measurement of Degree of Hydrolysis (DH)

The DH refers to the percentage of free amino terminal groups cleaved from proteins during hydrolysis and was determined using OPA (o-phthaldialdehyde) according to the method of Nielsen et al. [21] and Mirzaei et al. [22] with minor modifications. The content of the alpha-amino groups of the samples was determined as concentration of L-serine from a standard curve. The DH was then calculated as the ratio of alpha-amino nitrogen to total nitrogen content using the following equation:

(1) $$\begin{array}{c} DH\left(\;\%\;\right)=\frac{L_{i}-L_{0}}{L_{total}-L_{0}}\times 100, \end{array}$$

where *L*_*i*_ is the amount of released free amino groups resulting from hydrolysis at a time “*i*,” *L*_0_ is the amount of free amino groups in the original sample before the hydrolysis, and *L*_total_ is the total amount of free amino groups in the original sample following complete hydrolysis with 6 M HCl at 110°C for 24 h.

### 2.3. 2,2-Diphenyl-1-picrylhydrazyl (DPPH) Assay

The 2,2-diphenyl-1-picrylhydrazyl free radical (DPPH^•^) scavenging activity was measured according to the method described by Hwang et al. [23] with minor modification. 100 *μ*L aliquot of sample (1% w/v) was mixed with 100 *μ*L of 0.25 mM DPPH that was dissolved in 100% methanol. After incubation in the dark at 25°C for 30 min, the absorbance of the mixture was determined at 517 nm in a 96-well microplate reader (Labomed, model UVD-2950, Culver City, CA, USA). The following equation was used to calculate the percentage DPPH^•^ scavenging activity of the SHs:

(2) DPPH•  scavenging  activity%=1−As–AbAc×100,

where *A*_*s*_ is the absorbance of the tested stone fish hydrolysates (SHs), *A*_*b*_ is the absorbance of the blank, and *A*_*c*_ is the absorbance of the DPPH solution. All experiments were conducted in triplicate.

### 2.4. Ferrous Ion- (Fe^2+^-) Chelating Activity Assay

The Fe^2+^-chelating activity was determined as previously described by Carter [24] and Zarei et al. [25] with some modifications. A 10 *μ*L aliquot of 1% w/v SHs was mixed with 250 *μ*L of sodium acetate buffer (0.1 M, pH 4.9) and 30 *μ*L of 0.01% w/v, FeCl_2_ solution. Afterwards, 12.5 *μ*L of 0.04 M ferrozine solution was added following incubation for 30 min at 25°C and the absorbance was measured at 562 nm in a microplate reader. All determinations were carried in triplicate and the Fe^2+^-chelating effect was evaluated as follows:

(3) $$\begin{array}{c} {Fe}^{2+}\text{-chelating activity}\left(\;\%\;\right)=\left[\;1-\frac{\left(A_{s}-A_{b}\right)}{A_{c}}\;\right]\times 100, \end{array}$$

where *A*_*s*_ is the absorbance of the tested stone fish hydrolysates (SHs), *A*_*b*_ is the absorbance of the blank, and *A*_*c*_ is the absorbance of the control. All experiments were conducted in triplicate.

### 2.5. Effect of Shear Rate and Concentration on the Viscosity of SHs

Stone fish protein hydrolysates solutions were produced by dispersion in deionised water at varying concentrations (1, 2, 5, and 10% w/v respectively) under constant stirring for 60 min at 250 rpm and 40°C. A dynamic controlled stress (RS 600) rheometer model was used to study the effect of shear rate and concentration on the viscosity of the hydrolysates. The samples were equilibrated for 120 seconds between cone and plate configured with a diameter: 35 mm, cone angle: 2° and gap size: 0.5 mm to allow for the relaxation of residual stresses prior to testing. The samples were continuously sheared at a rate of 0 to 100 s^−1^ in 150 s. All determinations were made at 25°C and the following Ostwald-de Waele power law model equation was used to analyse the flow behavior of the hydrolysates:

(4) $$\begin{array}{c} \tau =K*{\dot{Y}}^{n}, \end{array}$$

where *τ* is the shear stress (Pa), $\dot{Y}$ is the shear rate (1/s), *K* is the consistency coefficient (Pa s^*n*^), and *n* is the flow behavior index.

### 2.6. Experimental Design for Model Building and Statistical Analysis

Minitab version 16.0 was used for the model building and statistical analysis according to previous description by Auwal et al. [20]. The individual and interaction effects of the process variables including pH, *X*_1_; temperature, *X*_2_; E/S ratio, *X*_3_; and time, *X*_4_ on the outcome responses; DH (*Y*_1_), DPPH^•^ scavenging activity (*Y*_2_), and Fe^2+^-chelating activity (*Y*_3_) were studied by RSM. Each response represents an average of experimental triplicates. A central composite design (CCD) was adapted with 31 experimental runs including 16 full factorial designs, 7 central points, and 8 axial points. The following second-order equation or its reduced form was used to fit the model:

(5) $$\begin{array}{c} Y=b_{0}+\overset{4}{\underset{i=1}{\sum }}b_{i}X_{i}+\overset{4}{\underset{i=1}{\sum }}b_{ii}{X_{i}}_{}^{2}+\overset{4}{\underset{i<j=2}{\sum }}b_{ij}X_{i}X_{j}, \end{array}$$

where *Y* is the outcome or dependent variable; *b*_0_ stands for the intercept while *b*_*i*_, *b*_*ii*_, and *b*_*ij*_ represent the coefficients of the linear, quadratic, and interaction terms whereas *X*_*i*_ and *X*_*j*_ are the independent or process variables, respectively. The statistical significance of the regression coefficients was evaluated using ANOVA. The fitted values predicted by the response regression equation were compared with the experimental values for validation of the model. Three-dimensional response surface plots were drawn using the Minitab version 16.0 to show the relationship between levels of the process variables and the outcome or response.

## 3. Results and Discussion

The observed effects due to the hydrolysis variables including pH (*X*_1_), temperature (*X*_2_), E/S ratio (*X*_3_), and time (*X*_4_) on the response values for DH (*Y*_1_), DPPH^•^ scavenging activity (*Y*_2_), and Fe^2+^-chelating activity (*Y*_3_) are shown in Table 1. The results for the model's coefficients of variation are given in Table 2. A probability test of *p* < 0.05 was used to estimate the statistical significance of variation in the observed responses using ANOVA. Other statistical parameters including coefficient of determination *R*^2^ (*R*-sqd), adjusted coefficient of determination *R*^2^-adjusted (*R*^2^-adj),* F*-test probability, and lack of fit values are also given in Table 2. The results for the *R*-sqd values of *Y*_1_, *Y*_2_, and *Y*_3_ are 98.50%, 98.14%, and 90.17% while the *R*^2^-adj values include 97.74%, 97.57%, and 87.17% for *Y*_1_, *Y*_2_, and *Y*_3_. The* F*-values of *Y*_1_, *Y*_2_, and *Y*_3_ are 131.01, 173.28, and 30.12 while the results for the lack of fit values of *Y*_1_, *Y*_2_, and *Y*_3_ are 0.076, 0.176, and 0.146, respectively (Table 2).

The large coefficient of determination (*R*^2^) and nonsignificant lack of fit values (*p* > 0.05) of *Y*_1_, *Y*_2_, and *Y*_3_ responses demonstrated the significance of the models and fitness of the experimental values to the theoretical values predicted by the model's regression equation (Table 2). The adjusted coefficient of determination (*R*^2^-adj) showed that the observed data variation of 97.74%, 97.57%, and 87.17% for *Y*_1_, *Y*_2_, and *Y*_3_ occurred due to the effects of the process conditions. The Fisher test (*F*-test) revealed high* F*-values and low* p* values of *p* < 0.05, which further validated the suitability of the models to the experimental data.

### 3.1. Degree of Hydrolysis (DH)

**Figure 1:**
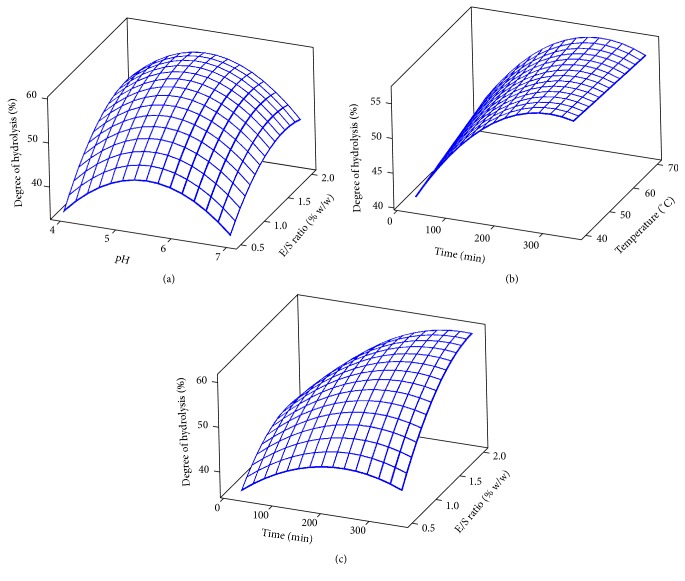
3D plots for degree of hydrolysis: (a) E/S ratio and pH; (b) temperature and time; (c) E/S ratio and time.

As shown in Table 2, a strong linear (*p* < 0.05) and quadratic (*p* < 0.05) effects of the hydrolysis variables in terms of pH, temperature, E/S ratio, and time were observed on DH as revealed by the regression coefficients. Hydrolysis temperature had no significant quadratic effect (*p* < 0.05) on DH. The three-dimensional (3D) response surface plots of DH are shown in Figure 1. The DH of stone fish protein was found to increase with the time of bromelain hydrolysis (Figure 1). As previously reported, the antioxidant activity is affected by the type of hydrolysis enzyme and the DH [14, 26, 27]. Ghanbari et al. [5] also observed higher DH of stone fish protein with increase in duration of hydrolysis for a total of 24 h.

### 3.2. 2,2-Diphenyl-1-picrylhydrazyl Radical (DPPH^•^) Scavenging Activity

**Figure 2:**
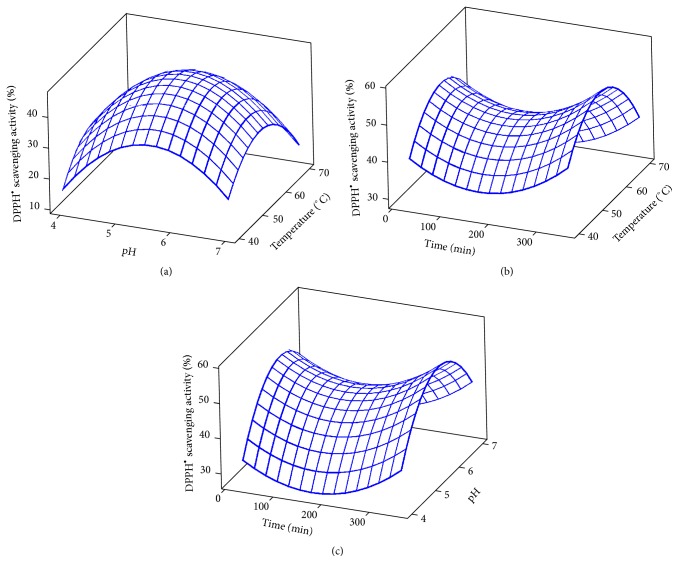
3D plots for DPPH radical-scavenging activity: (a) temperature and pH; (b) temperature and time; (c) pH and time.

The results for the DPPH^•^ scavenging activity are shown in Table 1 and Figure 2. The hydrolysis pH, temperature, and time indicated a strong linear (*p* < 0.05) and quadratic effects (*p* < 0.05) of the model with respect to DPPH^•^ scavenging activity. However, the hydrolysis E/S ratio exerted only linear effect on DPPH^•^ scavenging activity (Table 2). The 3D plots for the DPPH^•^ scavenging activity are given in Figure 2. The ability of stone fish protein hydrolysates to scavenge free radicals could be due to their potential to donate hydrogen and neutralise or stabilise free radicals and terminate their propagation. The effect might also be attributed to specific amino acid sequences in the hydrolysates typically hydrophobic amino acids and histidine, whose imidazole ring can potentially chelate free radicals and trap lipid. This is possible by forming a physical barrier around fat droplets which delay lipid oxidation and prevents free radical chain reaction [27–29]. As earlier reported by Zarei et al. [26], the hydrolysates derived from palm kernel cake protein exhibited DPPH^•^ scavenging activity that increased by increasing the protein hydrolysis time.

### 3.3. Ferrous Ion- (Fe^2+^-) Chelating Activity

**Figure 3:**
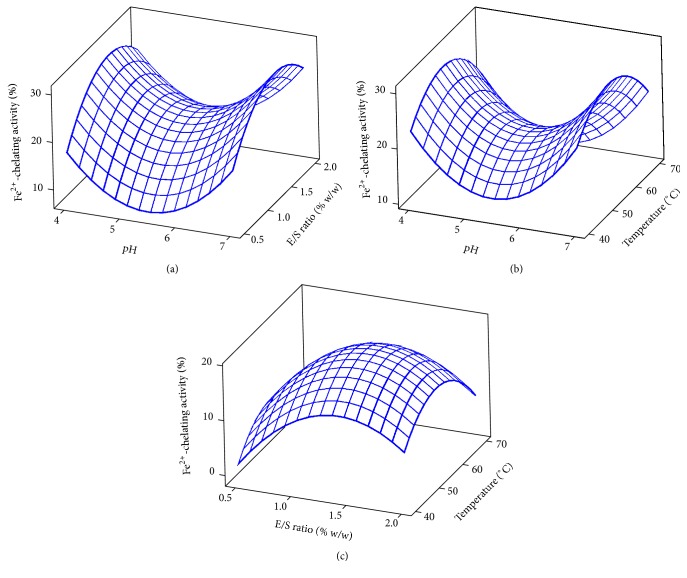
3D plots for Fe^2+^-chelating activity: (a) E/S ratio and pH; (b) temperature and pH; (c) temperature and E/S ratio.

The results for the Fe^2+^-chelating activity of stone fish protein hydrolysates are shown in Table 1 and Figure 3. The coefficients of regression revealed strong effects for both linear (*p* < 0.05) and quadratic (*p* < 0.05) terms of the hydrolysis pH, temperature, and E/S ratio on Fe^2+^-chelating activity. The hydrolysis time indicated no significant (*p* < 0.05) effect on Fe^2+^-chelating activity of stone fish protein hydrolysates (Table 2). The 3D plots for the Fe^2+^-chelating activity are shown in Figure 3. The initiation of oxidative chain reactions may result from generation of the first few radicals through the catalytic action of transition metals. The availability of transition metals can be reduced by chelating agents; thereby inhibiting the free radical-induced lipid peroxidation in both food and living systems, thus enhancing animal health as well as food stability safety and quality [30, 31].

Transition metal ions acts as electron donors and react very quickly with peroxides resulting in alkoxyl radical formation [31].

The result obtained showed that the peptides in stone fish protein hydrolysates exhibited Fe^2+^-chelating activity and can potentially reduce lipid oxidation.

### 3.4. Effect of Shear Rate and Concentration on the Viscosity of SHs

**Figure 4:**
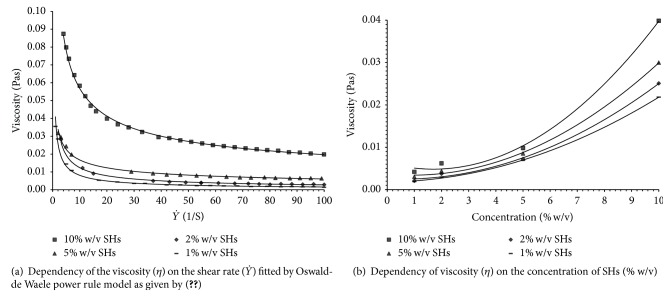
Dependency of viscosity on the shear rate (1/S) and concentration of SHs (% w/v).

The flow properties of the hydrolysates (Figure 4(a)) revealed a decrease in apparent viscosity (*η*) of all concentrations with increasing shear rate ($\overset{`}{Y}$), which might be due to the deformation within the flow field [32]. The higher shear rate at increasing concentration was also associated with the greater intermolecular interaction between the amino acids composition of the hydrolysates.

The flow curve of each concentration was fitted using the power rule (see (4)) [33–36]. The SHs demonstrated non-Newtonian behavior (*n* < 1) or pseudoplasticity.

In addition, the effects of four different concentrations (1, 2, 5, and 10% w/v) were studied on the viscosity of SHs solutions at different speed using the same dynamic shear rheometer. As shown in Figure 4(b), the flow behavior of the hydrolysates at increasing concentration and fixed shear rates indicated their stronger shear-thinning effect and higher viscosities.

The curves are widely separated from each other at higher concentrations and closely related to one another at lower concentration where the viscosities lie much closer (Figure 4(b)). The figure has clearly showed that the SHs undergone conformational changes from being flexible to a more rigid structure with increase in concentration. Hence, the higher viscosities obtained at 10% w/v could be due to the formation of larger molecular weight assemblies or aggregates of the hydrolysates as a result ofinteraction between the constituent peptides fragments. However, the viscosity appears to be independent of the speed or shear rate at lower concentration. This is evident by the smaller change in viscosities at these concentrations under the different shear rates [33].

Shear induces the breakage of particles lumps or aggregates to promote their flow at a particular shear stress. Shear thinning is also mediated by the removal of solvent layers from dissolved molecules, thereby decreasing the intermolecular interactions, and reduces flow resistance [37]. The pseudoplastic property or shear-thinning flow behavior of the SHs was found to vary over the ranges of selected shear rates.

At low shear rate, the effect of the initial shear orientation does not influence the Brownian motion that set all the particles at random. The pseudoplastic SHs solutions exhibited zero shear that was independent of shear rate and behaves similar to Newtonian liquids.

As the shear rate increases, the shear induced particles orientation that exceeds the random effect of Brownian motion and the viscosity decreased drastically. The viscosity decreased with further shear rate until it reached an asymptotically finite constant level beyond which no more shear thinning occurs with higher shear rate and the optimum of perfect orientation is attained [37].

Thus, the viscosity of the non-Newtonic or the pseudoplastic hydrolysates was independent of shear rate at both low (first Newtonian) and high (second Newtonian) ranges.

### 3.5. Optimisation of Hydrolysis Conditions

**Figure 5:**
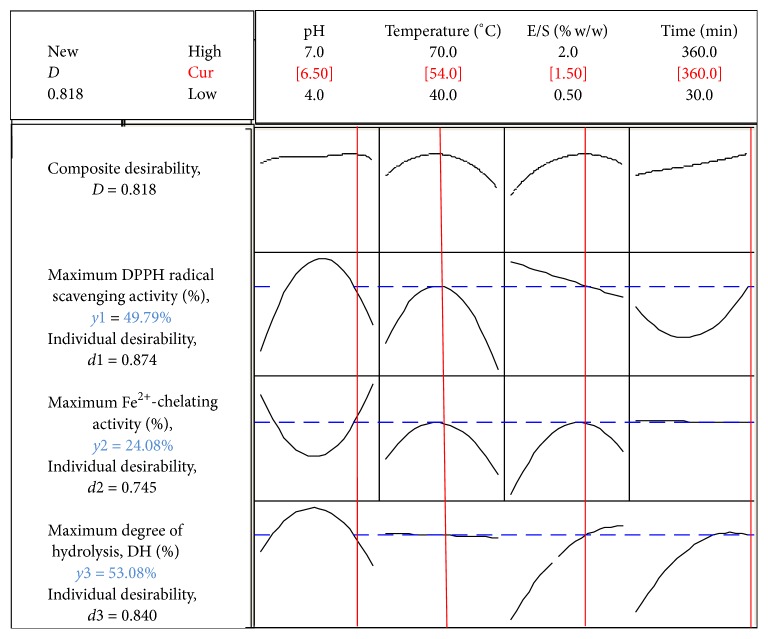
Response optimisation parameters, maximum predicted responses, and desirability levels.

Response surface optimiser (Figure 5) was used to determine the optimum conditions for the hydrolysis of stone fish protein to produce hydrolysates with maximum responses in terms of DPPH^•^ scavenging activity and Fe^2+^-chelating activity. The optimum levels obtained for the hydrolysis conditions were pH (6.5), temperature (54°C), E/S ratio (1.5% w/w), and time (360 min), respectively. The results for DH (54.62%) as well as DPPH^•^ scavenging activity (48.94%) and Fe^2+^-chelating activity (25.12%) obtained under these conditions were not statistically different from the predicted values of DH 53.08%; DPPH^•^ scavenging activity 49.79%; and Fe^2+^-chelating activity 24.08%, respectively, within 95% confidence interval.

Antioxidants act by absorbing free radicals and chelating metals to suppress lipid peroxidation [38]. Consequently, the antioxidant properties of stone fish protein hydrolysates can be exploited as a valuable functional ingredient in food formulation to improve dietary intake of antioxidants. Moreover, the hydrolysates can serve as a natural and safe additive to enhance the shelf-life of various food products that are susceptible to oxidation.

## 4. Conclusions

In this study, the optimal conditions of bromelain for the hydrolysis of stone fish protein in terms of pH, temperature, E/S ratio, and time were established for maximum responses of DPPH radical-scavenging activity and Fe^2+^-chelating activity using RSM. The optimised levels of these hydrolysis conditions were found to be pH 6.5, temperature 54°C, E/S ratio 1.5% w/w, and time 360 min. Under these conditions, the optimum values obtained for DH 54.62%, DPPH radical-scavenging activity 48.94%, and Fe^2+^-chelating activity 25.12% were not statistically different from the predicted values of DH 53.08%, DPPH radical-scavenging activity 49.79%, and Fe^2+^-chelating activity 24.08%, respectively, within 95% confidence interval.

## Figures and Tables

**Table 1:** Experimental and predicted values of response variables for central composite design.

Run order	^*∗*^Independent variables				^*∗∗*^Dependent variables					
	*X*_1_	*X*_2_	*X*_3_	*X*_4_	*Y*_1_ (DH, %)		*Y*_2_ (DPPH^•^ scavenging activity, %)		*Y*_3_ (Fe^2+^-chelating activity, %)	
	Uncoded levels				Experimental	Predicted	Experimental	Predicted	Experimental	Predicted
(1)	4.0	70	2.00	30	34.49	36.34	13.76	13.26	13.86	16.04
(2)	5.5	55	1.25	195	56.66	54.64	47.26	45.99	16.96	18.57
(3)	5.5	55	0.50	195	42.10	43.68	47.62	49.57	7.48	7.44
(4)	4.0	70	0.50	360	29.96	31.17	22.98	24.67	10.05	8.91
(5)	5.5	55	1.25	360	57.82	55.52	54.98	56.22	19.37	18.40
(6)	7.0	55	1.25	195	43.00	44.19	30.56	32.69	29.77	30.17
(7)	7.0	40	0.50	360	32.25	32.41	33.18	34.97	14.80	13.13
(8)	7.0	40	0.50	30	23.96	24.35	32.92	30.72	15.54	13.48
(9)	4.0	70	0.50	30	28.25	28.03	21.55	20.41	11.26	9.25
(10)	4.0	40	2.00	360	51.99	53.34	23.52	22.74	17.75	18.18
(11)	5.5	55	1.25	195	54.81	54.64	44.98	45.99	19.02	18.57
(12)	7.0	70	0.50	30	29.25	28.43	26.82	25.49	8.76	10.99
(13)	4.0	40	0.50	360	31.89	32.00	32.55	29.90	10.98	11.39
(14)	5.5	40	1.25	195	51.86	53.83	33.65	34.47	14.32	12.84
(15)	5.5	70	1.25	195	57.56	55.45	27.82	29.24	8.64	10.35
(16)	5.5	55	1.25	195	53.56	54.64	47.26	45.99	18.84	18.57
(17)	5.5	55	2.00	195	58.64	56.20	39.95	42.41	13.95	14.23
(18)	5.5	55	1.25	195	53.95	54.64	46.65	45.99	18.14	18.57
(19)	5.5	55	1.25	195	54.05	54.64	46.86	45.99	16.40	18.57
(20)	7.0	40	2.00	360	50.62	49.13	29.05	27.82	18.43	19.92
(21)	5.5	55	1.25	30	41.98	43.41	50.96	51.96	23.20	18.75
(22)	4.0	40	0.50	30	25.03	23.95	24.50	25.64	9.91	11.74
(23)	7.0	70	0.50	360	32.90	31.57	27.91	29.75	8.21	10.65
(24)	4.0	70	2.00	360	51.25	52.50	17.20	17.52	18.01	15.69
(25)	7.0	40	2.00	30	26.69	28.06	22.26	23.56	20.00	20.26
(26)	7.0	70	2.00	30	32.65	32.14	18.42	18.34	19.37	17.78
(27)	4.0	40	2.00	30	34.68	32.26	16.68	18.48	17.75	18.52
(28)	4.0	55	1.25	195	48.14	46.09	27.49	27.61	28.60	28.43
(29)	5.5	55	1.25	195	53.82	54.64	49.18	45.99	16.16	18.57
(30)	5.5	55	1.25	195	53.79	54.64	48.65	45.99	19.56	18.57
(31)	7.0	70	2.00	360	47.25	48.30	24.81	22.59	18.96	17.44

^*∗*^Independent variables for hydrolysis of stone fish protein: *X*_1_; pH, *X*_2_; temperature (°C), *X*_3_; enzyme/substrate ratio and *X*_4_; time (min). ^*∗∗*^Dependent variables: *Y*_1_; degree of hydrolysis (DH, %), *Y*_2_; 2,2-diphenyl-1-picrylhydrazyl radical (DPPH^•^) scavenging activity (%) and *Y*_3_; Fe^2+^-chelating activity (%).

**Table 2:** Regression coefficients, *R*^2^ and *F*-test probability for DH, DPPH^•^ scavenging activity, and Fe^2+^-chelating activity.

Factors	Coefficients					
	*Y*_1_	*p* value	*Y*_2_	*p* value	*Y*_3_	*p* value
Intercept	−116.530	0.000	−341.992	0.000	43.4458	0.005
*X*_1_	47.132	0.000	79.148	0.000	−51.8738	0.000
*X*_2_	0.151	0.003	6.735	0.000	3.3275	0.000
*X*_3_	29.774	0.000	−4.769	0.000	38.9285	0.000
*X*_4_	0.105	0.000	−0.103	0.000	−0.0010	0.709
*X*_1_ *∗X*_1_	4.226	0.000	−7.041	0.000	4.7686	0.000
*X*_2_ *∗X*_2_	−0.001	0.800	−0.063	0.000	−0.0310	0.000
*X*_3_ *∗X*_3_	−8.371	0.000	−1.484	0.487	−13.7613	0.000
*X*_4_ *∗X*_4_	−0.000	0.000	0.000	0.000	0.0001	0.077
*X*_1_ *∗X*_3_	−1.024	0.016	0.229	0.592	0.2378	0.573
*X*_2_ *∗X*_4_	−0.000	0.010	−0.000	0.222	0.0001	0.671
*X*_3_ *∗X*_4_	0.026	0.000	0.006	0.114	0.0018	0.635
*R* ^2^ (*R*-sqd)	98.50%		98.14%		90.170%	
*R* ^2^-adj	97.74%		97.57%		87.17%	
Lack of fit		0.076		0.176		0.146
*F*-values	131.01	0.000	173.28	0.000	30.12	0.000

## Abbreviations

SHs: Stone fish protein hydrolysates
RSM: Response surface methodology
CCD: Central composite design
OPA: O-Phthaldialdehyde
DH: Degree of hydrolysis
DPPH: 2,2-Diphenyl-1-picrylhydrazyl
ANOVA: Analysis of variance.
